# Supplementary material for: Causal Relationship between Aging and Anorexia Nervosa: A White-Matter-Microstructure-Mediated Mendelian Randomization Analysis
Source: Biomedicines. 2024 Aug 16;12(8):1874. doi: 10.3390/biomedicines12081874 (PMC11351342; doi:10.3390/biomedicines12081874)
Supplement: Supplementary file 1 [file biomedicines-12-01874-s001.zip › Supplementary Figure/Figure S4/Singe SNP plot of MR analysis for TL to ubm-b-1967.pdf]

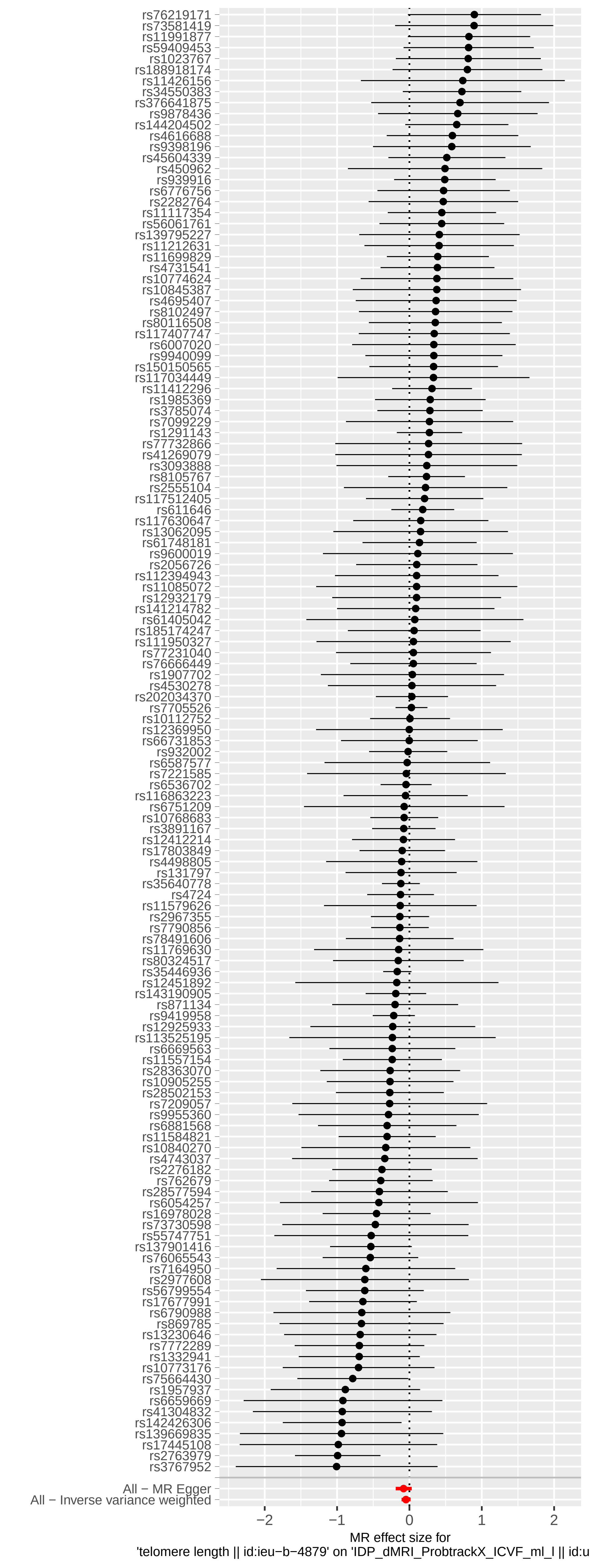

rs76219171
rs73581419
rs11991877
rs59409453
rs1023767
rs188918174
rs11426156
rs34550383
rs376641875
rs9878436
rs144204502
rs4616688
rs9398196
rs45604339
rs450962
rs939916
rs6776756
rs2282764
rs11117354
rs56061761
rs139795227
rs11212631
rs11699829
rs4731541
rs10774624
rs10845387
rs4695407
rs8102497
rs80116508
rs117407747
rs6007020
rs9940099
rs150150565
rs117034449
rs11412296
rs1985369
rs3785074
rs7099229
rs1291143
rs77732866
rs41269079
rs3093888
rs8105767
rs2555104
rs117512405
rs611646
rs117630647
rs13062095
rs61748181
rs9600019
rs2056726
rs112394943
rs11085072
rs12932179
rs141214782
rs61405042
rs185174247
rs111950327
rs77231040
rs76666449
rs1907702
rs4530278
rs202034370
rs7705526
rs10112752
rs12369950
rs66731853
rs932002
rs6587577
rs7221585
rs6536702
rs116863223
rs6751209
rs10768683
rs3891167
rs12412214
rs17803849
rs4498805
rs131797
rs35640778
rs4724
rs11579626
rs2967355
rs7790856
rs78491606
rs11769630
rs80324517
rs35446936
rs12451892
rs143190905
rs871134
rs9419958
rs12925933
rs113525195
rs6669563
rs11557154
rs28363070
rs10905255
rs28502153
rs7209057
rs9955360
rs6881568
rs11584821
rs10840270
rs4743037
rs2276182
rs762679
rs28577594
rs6054257
rs16978028
rs73730598
rs55747751
rs137901416
rs76065543
rs7164950
rs2977608
rs56799554
rs17677991
rs6790988
rs869785
rs13230646
rs7772289
rs1332941
rs10773176
rs75664430
rs1957937
rs6659669
rs41304832
rs142426306
rs139669835
rs17445108
rs2763979
rs3767952
All − MR Egger
All − Inverse variance weighted
−2
−1
0
1
2
MR effect size for
'telomere length || id:ieu−b−4879' on 'IDP_dMRI_ProbtrackX_ICVF_ml_l || id:u
